# Specific TCR profiles predict clinical outcome of adjuvant EGFR-TKIs for resected *EGFR*-mutant non-small cell lung cancer

**DOI:** 10.1186/s40364-023-00470-z

**Published:** 2023-03-07

**Authors:** Si-Yang Maggie Liu, Cunte Chen, Yi-Kai Zhang, Wen-Zhao Zhong, Yi-Long Wu, Si-Yang Liu, Yangqiu Li

**Affiliations:** 1 Department of Hematology, The First Affiliated Hospital, Jinan University, Guangzhou, 510632 China; 2 Key Laboratory for Regenerative Medicine of Ministry of Education, Institute of Hematology, School of Medicine, Jinan University, Guangzhou, 510632 China; 3 Guangdong Lung Cancer Institute, Guangdong Provincial People’s Hospital (Guangdong Academy of Medical Sciences), Southern Medical University, Guangzhou, 510080 China

**Keywords:** T-cell receptor repertoire, Vβ7-3Jβ2-5/Vβ24-1Jβ2-1/Vβ28Jβ2-2/Vβ5-6Jβ2-7, *EGFR* mutation, Adjuvant EGFR-TKI, Non-small cell lung cancer, CTONG1104

## Abstract

**Background:**

ADJUVANT-CTONG1104 reported a favorable survival outcome from adjuvant gefitinib treatment over chemotherapy in *EGFR*-mutant non-small cell lung cancer (NSCLC) patients. However, heterogeneous benefit from EGFR-TKIs and chemotherapy demands further biomarker exploration for patient selection. Previously, we identified certain TCR sequences with predictive value for adjuvant therapies from the CTONG1104 trial and found a relationship between the TCR repertoire and genetic variations. It remains unknown which TCR sequences could further enhance the prediction for only adjuvant EGFR-TKI.

**Methods:**

In this study, 57 tumor and 12 tumor-adjacent samples, respectively, from gefitinib-treated patients in the CTONG1104 were collected for TCR β gene sequencing. We attempted to constitute a predictive model for prognosis and favorable adjuvant EGFR-TKI outcome for patients with early-stage NSCLC and *EGFR* mutations.

**Results:**

The TCR rearrangements demonstrated significant prediction for overall survival (OS). A combined model of high frequent Vβ7-3Jβ2-5 and Vβ24-1Jβ2-1 with lower frequent Vβ5-6Jβ2-7 and Vβ28Jβ2-2 constituted the best value for predicting OS (*P* < 0.001; Hazard Ratio [HR] = 9.65, 95% confidence interval [CI]: 2.27 to 41.12) or DFS (*P* = 0.02; HR = 2.61, 95% CI: 1.13 to 6.03). In Cox regression analyses, when multiple clinical data were included, the risk score remained an independent prognostic predictor for OS (*P* = 0.003; HR = 9.49; 95% CI: 2.21 to 40.92) and DFS (*P* = 0.015; HR = 3.13; 95% CI: 1.25 to 7.87).

**Conclusions:**

In this study, a predictive model was constituted with specific TCR sequences for prognosis prediction and gefitinib benefit in the ADJUVANT-CTONG1104 trial. We provide a potential immune biomarker for *EGFR*-mutant NSCLC patients who might benefit from an adjuvant EGFR-TKI.

**Supplementary Information:**

The online version contains supplementary material available at 10.1186/s40364-023-00470-z.

**To the Editor,**

Adjuvant epidermal growth factor receptor-tyrosine kinase inhibitors (EGFR-TKIs) have shifted the treatment paradigm for patients with early-stage non-small-cell lung cancer (NSCLC) and *EGFR* mutations [1]. Promising progress has been achieved by prospective clinical trials, such as CTONG1104 and ADAURA [2, 3]. However, not all patients could obtain clinical benefits from adjuvant TKIs. Our previous study demonstrated that a portion of *EGFR*-mutant patients achieves different favorable benefits from adjuvant EGFR-TKIs and chemotherapy [4], which raises the necessity for further biomarker exploration.

The T cell receptor (TCR) repertoire plays an important role in the prognosis of cancer patients and could serve as a predictive biomarker for checkpoint blockade therapy [5, 6]. The clonality and diversity of TCRs on tumor-infiltrated lymphocytes (TILs) represent the tumor microenvironment status, which reflects the balance of the anti-tumor immunity in the host [7, 8]. In our previous publications, we identified certain TCR Vβ-Jβ sequences that had predictive value for prognoses and adjuvant therapies in patients with *EGFR-*mutant NSCLC from the ADJUVANT-CTONG1104 trial [9], and the potential mechanism may be due to gene mutation or abnormal amplification, which could induce T cell activation through clonal expansion of antigen-specific T cells [10]. However, whether there is a predictive model that combines TCR sequences with positive and negative values that could further enhance the prediction of prognosis and adjuvant EGFR-TKI treatment is unknown.

In this study, we investigated a biomarker with a specific TCRβ combination for the prediction of prognosis and benefit from adjuvant gefitinib in *EGFR*-mutant NSCLC patients from the ADJUVANT-CTONG1104 trial. Fifty seven resected tumor samples and 12 tumor-adjacent samples from gefitinib-treated patients were collected for TCRβ gene sequencing to obtain TCR repertoires (Online Materials and Methods). In total, there were 89 and 105 differentially expressed TCRs that were identified with low and high clonality, respectively (fold-change > 1.2, *P* < 0.05) (Fig. 1A). Univariate COX regression analysis was performed in the high clonality group (frequency > 0.1%), where three TCR rearrangements demonstrated statistical significance in predicting poor overall survival (OS), and five TCRs demonstrated good prediction for OS (*P* < 0.1) (Fig. 1B). Ultimately, the combination of higher frequent Vβ7-3Jβ2-5 and Vβ24-1Jβ2-1 with lower frequent Vβ5-6Jβ2-7 and Vβ28Jβ2-2 constituted the best model for predicting OS after internal validation using 100 repeated 10-fold cross validations (Fig. 1C). Among these four TCRs, Vβ7-3Jβ2-5 and Vβ24-1Jβ2-1 had the highest contribution in the multivariate COX regression model, which was associated with good and poor prognosis, respectively (Fig. 1D).

**Fig. 1:**
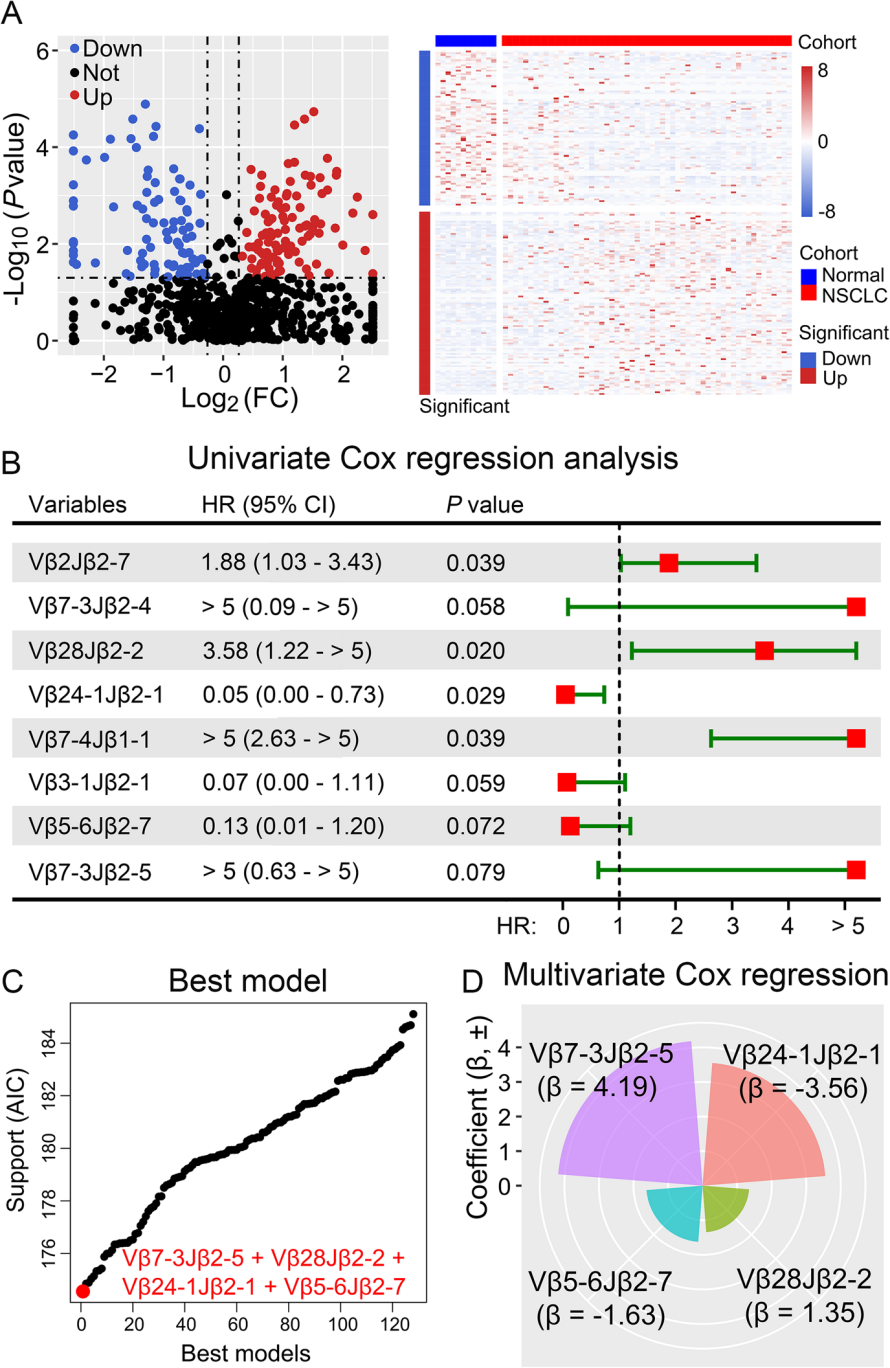
Identification of combined TCRs could predict the prognosis of adjuvant gefitinib. Identification of differentially expressed TCRs by the Mann-Whitney-Wilcoxon test based on tumor vs. tumor-adjacent tissue (**A**). Forest plot of univariate COX regression analysis showing the clonal TCRs associated with favorable overall survival (OS) based on *P* < 0.1 (**B**). The best resultant model that identified with four TCRs (**C**). The radar plot shows the contribution of the 4 TCRs to OS, which was determined by the coefficients of the 4 TCRs in the multivariate COX regression model (**D**)

Next, we evaluated the ability of this combination model to predict OS and disease-free survival (DFS) in *EGFR-*mutant patients who received adjuvant gefitinib therapy. The risk score was negatively related to survival benefit, regardless of OS or DFS. The survival curves were significantly separated, and 90% of the patients with a low-risk score survived for 5 years (Fig. 2A). Further, we assessed the independent prediction of the four TCRs by Kaplan-Meier analysis. We reported Vβ24-1Jβ2-1 in our previous publication [9], which is consistent with the current results. Of note, patients with a high frequency of Vβ7-3Jβ2-5 had significantly poor OS and DFS (*P* < 0.01, Fig. 2B). A previous study by Han et al. demonstrated that TRBV7-3 disappeared in patients with pseudo-progression disease after checkpoint blockade therapy [11], suggesting that this TCR sequence may play an impaired role in anti-tumor immunity and might have similar predictive value on both immunotherapy and targeted therapy. However, it still needs further functional investigation. Next, we performed univariate and multivariate Cox proportional hazard regression analyses. When sex, age, smoking history, pathology, clinical stage, N stage, and risk score were included, the results suggested that the risk score was an independent prognostic predictor for OS and DFS (Supplementary Table 1).

**Fig. 2:**
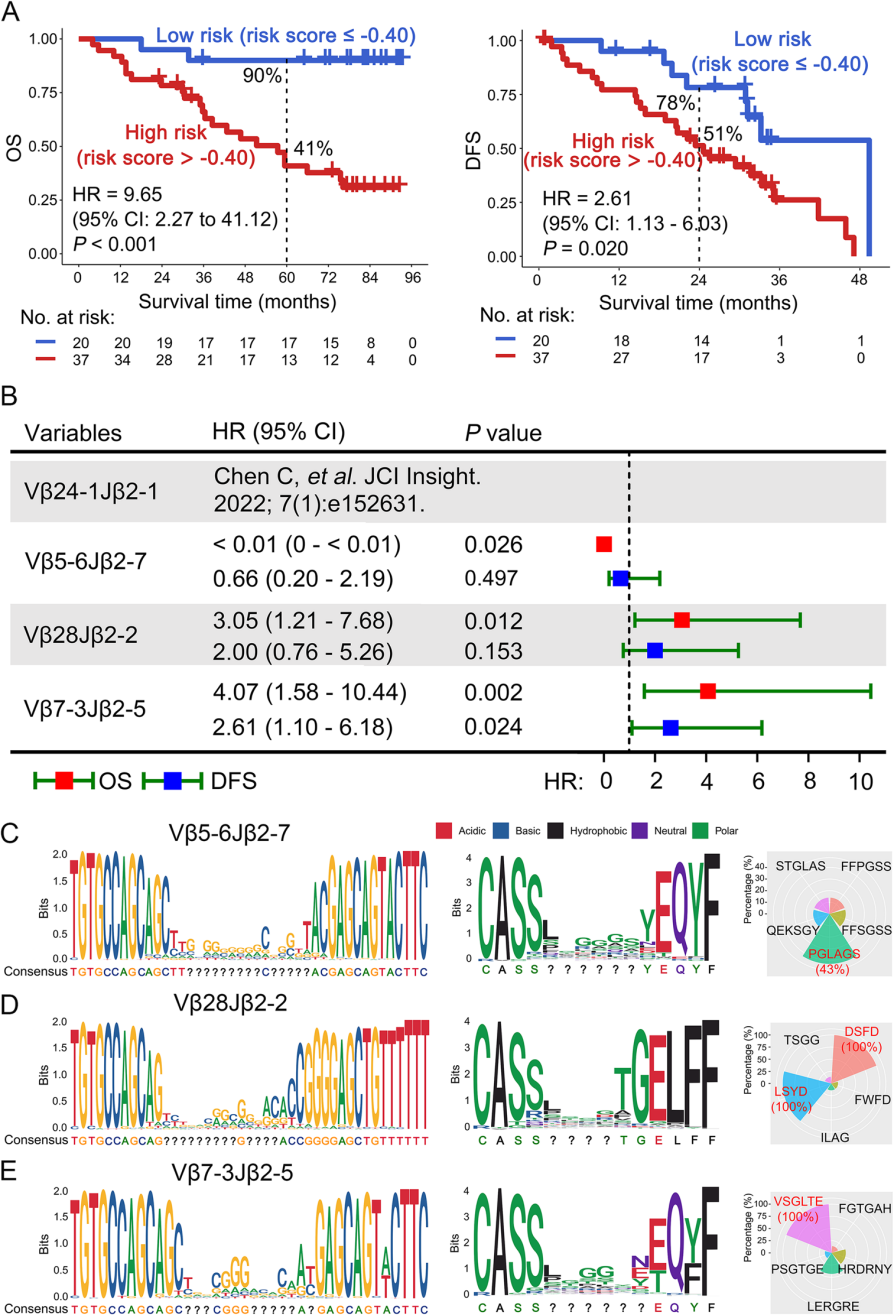
A weighted combination of four TCRs was associated with the prognosis of patients with an *EGFR* mutation with NSCLC. **A** Overall survival (left) and disease-free survival (right) analysis of low- and high-risk patients based on the combination of four TCRs. The risk score was negatively related to survival benefit regardless of OS or DFS (*P* < 0.001; Hazard Ratio [HR] = 9.65, 95% confidence interval [CI]: 2.27 to 41.12) or DFS (*P* = 0.02; HR = 2.61, 95% CI: 1.13 to 6.03). **B** Survival analysis of patients with a low and high frequency of Vβ5-6Jβ2-7, Vβ28Jβ2-2, and Vβ7-3Jβ2-5 by Kaplan-Meier analysis; Vβ24-1Jβ2-1 was published in our previous studies. Local alignment was used to calculate the similarity of the base (left) and amino acid (middle) sequences of Vβ5-6Jβ2-7 (**C**), Vβ28Jβ2-2 (**D**), and Vβ7-3Jβ2-5 (**E**). The proportion of the top 5 non-conservative amino acid sequences in patients with a high frequency of TCRs is also shown (right)

To confirm the TCR clonotypes, we explored the nucleotide (NT) and amino acid (AA) sequences and identified the top five CDR3 motifs in Vβ5-6Jβ2-7, Vβ28Jβ2-2, and Vβ7-3Jβ2-5 in the high-frequency TCR groups. The Vβ24-1Jβ2-1 results were published in our previous study [9]. The top CDR3 motif for Vβ5-6Jβ2-7 was PGLAGS with 43% frequency (Fig. 2C). For Vβ28Jβ2-2, the top CDR3 motifs were LSYD and DSFD with 100% frequency (Fig. 2D), and for Vβ7-3Jβ2-5, the top CDR3 motif was VSGLTE with 100% frequency (Fig. 2E). Overall, this study identified TCR CDR3 sequences that might be tumor antigen-specific TCRs in patients, which may be further used as biomarkers to predict the efficacy of adjuvant EGFR-TKIs.

In summary, we for the first time developed a predictive model with specific TCR profile sequences that could serve as a biomarker for the prediction of OS and DFS from adjuvant gefitinib in early-stage *EGFR*-mutant NSCLC patients in the ADJUVANT-CTONG1104 trial. This model is a potential immune biomarker for *EGFR*-mutant NSCLC patients who might obtain benefits from adjuvant EGFR-TKIs. However, limitations of the study include insufficient tissue availability for this retrospective analysis of all enrolled participants. In the future, an external validation cohort will be available by another trial result, and functional verification of the findings will be performed.

## Supplementary Information

**Additional file 1.** Supplementary online methods.

**Additional file 2: Table S1.** Univariate and multivariate COX regression analysis in patients with *EGFR*-mutant NSCLC with stage II/III.

## Data Availability

Please contact the corresponding author for data requests.
