# Supplementary material for: Specific TCR profiles predict clinical outcome of adjuvant EGFR-TKIs for resected EGFR-mutant non-small cell lung cancer
Source: Biomark Res. 2023 Mar 7;11:26. doi: 10.1186/s40364-023-00470-z (PMC9990191; doi:10.1186/s40364-023-00470-z)
Supplement: Supplementary file 1 — Additional file 1. Supplementary online methods. [file 40364_2023_470_MOESM1_ESM.doc]

**Materials and methods**

**Patients**

Archived formalin-fixed and paraffin-embedded (FFPE) blocks of tumor tissues and tumor-adjacent tissues were prospectively collected from 57 and 12 gefitinib-treated patients in the ADJUVANT-CTONG1104 trial for TCRβ gene sequencing [1, 2]. The demographic and clinical information were described in our previous publication [3].

**TCR sequencing and analysis**

DNA was extracted from FFPE samples and performed for multiplex polymerase chain reaction (PCR) for the construction of TCR libraries (QIAGEN Multiplex PCR Plus Kit). Purification of amplified synthetic products was then performed using the AxyPrep MAG FragmentSelect-I Kit (Axygen), and the KAPA Hyper Prep Kit (KAPA Biosystems) was used for library preparation. TCR libraries were then sequenced with the Illumina HiSeq 4000 platform.

Fastq files were processed to remove adapters and low-quality reads with trimmomatic for TCR profiling. Non-V-J paired reads were further removed by Cutadapt (V 1.18). Paired-end read merger (PEAR, V 0.9.10) was employed to merge paired reads and synthetic standards. The tumor samples were then cleaned using barcodes. MiXCR (V 2.1.11) was subsequently used for the assembly of clean reads, which were then aligned to reference V/J gene segments based on the international ImMunoGeneTics (IMGT) database. The TCR diversity and clonality were analyzed as previously described with the R package vegan [4].

**Statistical Analysis**

All statistical analyses were performed with R software (version 4.0.2). Univariate and multivariate Cox regression analyses were performed by the package "survival". The predictive model with best values was obtained by the package "glmulti". The optimal cutoff points for quantitative data were determined by maximally selected rank statistics in the package "maxstat". Kaplan-Meier analysis was used to compare survival curves with high and low risk scores. Finally, the consensus of nucleotide and amino acid sequences was aligned and calculated by the package "msa". A two-tailed P<0.05 was regarded as statistical significance.

The risk score = 4.19*(Vβ7-3Jβ2-5) +1.35*(Vβ28Jβ2-2) -3.56*(Vβ24-1Jβ2-1) -1.63*(Vβ5-6Jβ2-7).

**References:**

1. Zhong WZ, Wang Q, Mao WM, Xu ST, Wu L, Shen Y, et al. Gefitinib versus vinorelbine plus cisplatin as adjuvant treatment for stage II-IIIA (N1-N2) EGFR-mutant NSCLC (ADJUVANT/CTONG1104): a randomised, open-label, phase 3 study. Lancet Oncol. 2018;19:139-148.

2. Liu SY, Bao H, Wang Q, Mao WM, Chen Y, Tong X, et al. Genomic signatures define three subtypes of EGFR-mutant stage II-III non-small-cell lung cancer with distinct adjuvant therapy outcomes. Nat Commun. 2021;12:6450.

3. Chen C, Liu SM, Chen Y, Ou Q, Bao H, Xu L, et al. Predictive value of TCR Vβ-Jβ profile for adjuvant gefitinib in EGFR mutant NSCLC from ADJUVANT-CTONG 1104 trial. JCI Insight. 2022;7:e152631.

4. Chen C, Liu SM, Chen Y, Han M, Ou Q, Bao H, et al. Poor prognosis of intra-tumoural TRBV6-6 variants in EGFR-mutant NSCLC: Results from the ADJUVANT-CTONG1104 trial. Clin Transl Med. 2022;12:e775.
