# Supplementary material for: Specific TCR profiles predict clinical outcome of adjuvant EGFR-TKIs for resected EGFR-mutant non-small cell lung cancer
Source: Biomark Res. 2023 Mar 7;11:26. doi: 10.1186/s40364-023-00470-z (PMC9990191; doi:10.1186/s40364-023-00470-z)
Supplement: Supplementary file 2 — Additional file 2: Table S1. Univariate and multivariate COX regression analysis in patients with EGFR-mutant NSCLC with stage II/III. [file 40364_2023_470_MOESM2_ESM.docx]

**Table S1.** Univariate and multivariate COX regression analysis in patients with *EGFR*-mutant NSCLC with stage II/III.

| Variables | OS | | | | |  | DFS | | | | |
| --- | --- | --- | --- | --- | --- | --- | --- | --- | --- | --- | --- |
|  | Univariate COX | |  | Multivariate COX | |  | Univariate COX | |  | Multivariate COX | |
|  | HR  (95% CI) | *P* value |  | HR  (95% CI) | *P* value |  | HR  (95% CI) | *P* value |  | HR  (95% CI) | *P*  value |
| Risk score (reference low risk) | | |  |  |  |  |  |  |  |  |  |
| High risk | 9.61  (2.26, 40.92) | 0.002 |  | 9.49  (2.21, 40.88) | 0.003 |  | 2.61  (1.13, 6.03) | 0.025 |  | 3.13  (1.25, 7.87) | 0.015 |
| Sex (reference female) | | |  |  |  |  |  |  |  |  |  |
| Male | 1.17  (0.53, 2.59) | 0.696 |  | 1.58  (0.51, 4.91) | 0.430 |  | 1.32  (0.64, 2.71) | 0.449 |  | 1.46  (0.42, 5.14) | 0.553 |
| Age (yr) | 1.01  (0.96, 1.06) | 0.711 |  | 1.00  (0.96, 1.05) | 0.899 |  | 1.01  (0.97, 1.05) | 0.717 |  | 1.00  (0.96, 1.05) | 0.877 |
| Smoking history (reference never) | | |  |  |  |  |  |  |  |  |  |
| Ever | 1.19  (0.40, 3.55) | 0.752 |  | 1.73  (0.41, 77.26) | 0.452 |  | 1.00  (0.38, 2.64) | 0.995 |  | 1.16  (0.26, 5.06) | 0.848 |
| Current | 1.26  (0.43, 3.76) | 0.674 |  | 1.27  (0.33, 4.93) | 0.733 |  | 0.84  (0.29, 2.46) | 0.748 |  | 0.84  (0.17, 4.16) | 0.833 |
| Pathology (reference adenocarcinoma) | | | | |  |  |  |  |  |  |  |
| Other | 1.06  (0.25, 4.49) | 0.940 |  | 0.80  (0.17, 3.68) | 0.772 |  | 1.80  (0.62, 5.20) | 0.281 |  | 1.16  (0.36, 3.71) | 0.800 |
| Clinical stage (reference II) | | |  |  |  |  |  |  |  |  |  |
| III | 1.30  (0.52, 3.25) | 0.582 |  | 0.00  (0.00, > 50) | 0.955 |  | 1.88  (0.77, 4.61) | 0.168 |  | 4.24  (0.43, 41.99) | 0.217 |
| N stage (reference N1) | | |  |  |  |  |  |  |  |  |  |
| N2 | 1.46  (0.58, 3.66) | 0.419 |  | > 50  (0.00, > 50) | 0.952 |  | 1.75  (0.75, 4.08) | 0.197 |  | 0.53  (0.06, 4.62) | 0.566 |

CI: confidence interval; DFS: disease-free survival; HR: hazard ratio; OS: overall survival.
